# Molecular Analysis of Virulent Determinants of Enterovirus 71

**DOI:** 10.1371/journal.pone.0026237

**Published:** 2011-10-19

**Authors:** Renqing Li, Qinghua Zou, Lijuan Chen, Herun Zhang, Yumei Wang

**Affiliations:** 1 Beijing Center for Disease Control and Prevention, Beijing, China; 2 Department of Microbiology, Peking University Health Science Center, Beijing, China; University of Illinois at Chicago, United States of America

## Abstract

Enterovirus 71 (EV71) is the most important causative agent of hand, foot and mouth disease (HFMD) in children. In most cases, it is a self-limiting illness. However some EV71 infectious cases can develop severe clinical outcomes, such as encephalitis, meningitis, poliomyelitis like paralysis, and even death. To identify the determinants of virulence, the deduced amino acid sequence of polyprotein and nucleotide sequence of 5′-NTR and 3′-NTR in 25 SC-EV71 strains (strains from severe cases) and 31 MC-EV71 strains (strains from mild cases) were analyzed. Results showed four amino acids on two positions (Gly^P710^/Gln^P710^/Arg^P710^ and Glu^P729^) on the DE and EF loop of VP1, one (Lys^P930^) on the surface of protease 2A and four nucleotides on three positions (G^P272^, U^P488^ and A^P700^/U^P700^) in the 5'-NTR region are associated with EV71 virulent phenotype. Predicted secondary structure of RNA using the consensus sequence of 5'-NTR by RNAStructure showed the mutation of nucleotide at position 488 in strain BJ08-Z004-3 (position 491 in prototype strain BrCr) can result in the discrepancy of an additional pair of nucleotides and thus change the stability of the second structure of IRES. Fragment base content analysis showed that in the region 696 to 714 bp at the 5'-NTR, where the A^P700^/U^P700^ was located, the nucleotide constitution ratios differed significantly between SC-EV71 and MC-EV71 strains. In conclusion, comparative genomic analysis showed that virulence of EV71 strains are mainly determined by the amino acids on two positions of VP1, one position of protease 2A and the nucleotides on three positions in 5'-NTR.

## Introduction

Enterovirus 71 is one of the enteroviruses that are most often associated with large outbreaks of hand, foot and mouth disease (HFMD) [1]. In most cases, it is a self-limiting illness. However over the last decade fatal cases with symptoms of central nervous system (CNS) involvement emerged during the HFMD outbreaks in Malaysia [2], Taiwan [3], Australia [4], South Korea [5], Japan [6], Singapore [7] and Vietnam [8]. In the mainland of People's Republic of China, a total of 488,955 HFMD cases were reported nationwide in 2008, including 126 fatal cases. The number of HFMD cases reported increased to 1,155,525 in 2009, including 353 fatal cases which were almost all due to EV71 infection (http://www.moh.gov.cn/publicfiles/business/htmlfiles/mohbgt/s3582/201002/46043.htm). It has been postulated that differences in the clinical outcomes associated with EV71 infection are possibly due to differences in the virulence of the virus [9].

The genome of EV71 contains a single-stranded positive genomic RNA of about 7,400 bp in length. The single open reading frame (ORF) encodes a polyprotein and is flanked by non-translated regions (NTR) at the 5′ and 3′ end [9]. The polyprotein can be divided into three genomic regions (P1, P2 and P3). The P1 region encodes the capsid protein comprised of four structural proteins VP1–4. The P2 and P3 region encode the nonstructural proteins including 2A–C, 3A–D [1]. Functionally the high structured 5′-NTR of enterovirus like poliovirus can be divided into three regions: the 5′ terminal cloverleaf (1 to 89–101 bp), internal ribosome entry site (IRES) element (123–126bp to 602–605bp) and hypervariable region (last 120bp) [10]. The IRES element controls the translation of the viral mRNA [11]. It has been reported that sequences in the 5′-NTR and viral RNA-dependent RNA polymerase 3D gene are important in determining the neurovirulence of polioviruses [12], [13]. Mutation of the EV71 standard strain BrCr in the 5′-NTR and 3D region showed attenuated neurovirulence in the cynomolgus monkey model [14]. In enterovirus, the 3′-NTR is a highly conserved domain and point mutations can result in a lethal phenotype [15], [16]. Other researches also investigated the virulence determinants of EV71 strains [17]. However because the strains analyzed are limited, the results are not representative. With more and more EV71 genomes being completed and uploaded to Genbank database, it is possible to have a clearer investigation about the virulence determinants in the genome. In this study, genome sequences of five EV71 strains with different clinical outcomes isolated in our laboratory and 51 EV71 strains with complete genome sequences and identified clinical backgrounds in Genbank database are analyzed. Determinants for virulence were explored.

## Results

### Phylogenetic analysis of VP1 coding region and the complete polyprotein coding region

**Figure 1:**
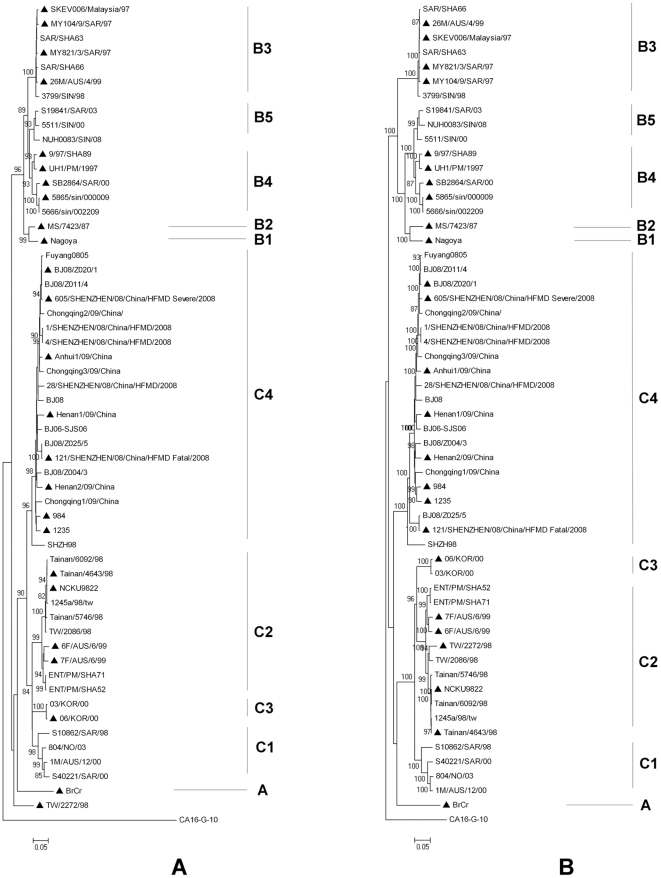
Phylogenetic analysis of VP1 protein coding region and the complete polyprotein coding region of EV71 strains. A: phylogenetic tree based on VP1 protein coding region. B: phylogenetic tree based on polyprotein coding region. ▴: SC-EV71 strain.

Phylogenetic analysis of our five EV71 strains and 51 EV71 strains with identified backgrounds from GenBank database was performed by neighbor-joining method based on VP1 coding region (891 nucleotides) or complete polyprotein coding region (6582 nucleotides). Results showed the 25 SC-EV71 and 31 MC-EV71 strains can be divided into three distinct genotypes A, B (including B1 to B5) and C (C1 to C4) (Figure 1). Results based on VP1 coincide with results based on polyprotein perfectly, except strain TW/2272/98, which was isolated from a patient died of pulmonary hemorrhage and shock in Taiwan in 1998. This strain was grouped as subgenotype C2 based on polyprotein, but was subsequently repositioned out of genotype B and genotype C when tested by VP1. Our five strains were all subdivided into C4 subgenotype in the phylogenetic trees (Figure 1).

### Relationship between virulence and genotype

The distribution of SC-EV71 and MC-EV71 strains in different genotypes was analyzed to see whether there are some correlations between virulence and genotype. Almost all the strains fall into either genotype B or C, except the prototype BrCr virus (ETU22521.1). The rates of SC-EV71 strains in the two genotypes are 58.8% (10/17) and 36.8% (14/38) respectively, and there is no significant difference between them (χ2 = 2.302, p = 0.129, α = 0.05) (Table 1). So we found no genotypes correlated with EV71 virulence.

**Table 1:** The number of SC-EV71 and MC-EV71 strains in different genotypes.

genotype	A	B					C				Total
		B1	B2	B3	B4	B5	C1	C2	C3	C4	
MC-EV71 strains	0	0	0	3	1	3	4	6	1	13	31
SC-EV71 strains	1	1	1	4	4	0	0	5	1	8	25
Total	1	1	1	7	5	3	4	11	2	21	56

### Analysis of the amino acids of polyprotein in SC-EV71 and MC-EV71 strains

Deduced amino acid sequences of polyprotein of the 25 SC-EV71 and 31 MC-EV71 strains are aligned. All the sequences are the same in length, and about 93.6% of the amino acids are the same in the 56 strains. Positions where amino acids are different in more than two strains are defined as variant positions. 139 such positions are found. The distribution of the positions can be found in Table 2. In structural protein VP4, VP2 and VP3, amino acid variation rates are low (0–2.9%) with the lowest in VP4 (0%). While in non-structural proteins 2B, 3A, 3B, 3C and 3D, amino acid variation rates are high (9.1%–10.9%) with the highest in 3C (10.9%). In the middle region of polyprotein, such as VP1, 2A and 2C, the substitution rates are in the medium (4.7%–6.4%).

**Table 2:** The distribution of variant amino acid positions in EV71 proteins.

Protein	Number of variant positions	Rate*
VP4	0	0% (0/69)
VP2	6	2.4% (6/254)
VP3	7	2.9% (7/242)
VP1	14	4.7% (14/297)
2A	8	5.3% (8/150)
2B	9	9.1% (9/99)
2C	21	6.4% (21/329)
3A	8	9.3% (8/86)
3B	2	9.1% (2/22)
3C	20	10.9% (20/183)
3D	44	9.5% (44/462)

*: rate = number of variant positions/total number of amino acids of the protein.

In these variant positions, the constituent ratios of variant residues in three positions appear significantly different between SC-EV71 and MC-EV71 strains. On position 710 (P710), the constituent ratios of four residues (Glu/Gly/Gln/Arg) in SC-EV71 strains are differed from those in MC-EV71 strains (χ2 = 9.304,p = 0.011, α = 0.05). For the three amino acids Gly, Gln and Arg on P710, it is more likely to appear in SC-EV71 strains than in MC-EV71 strains (χ2 = 7.139, p = 0.008, α = 0.05). On P729, there are two residues (Asp/Glu). The constituent ratio of Glu in SC-EV71 strains is higher than in MC-EV71 strains (χ2 = 4.021, p = 0.045, α = 0.05). On P930, there are three residues (Met/Arg/Lys). The constituent ratio of Lys is higher in SC-EV71 strains than in MC-EV71 strains (χ2 = 3.976, p = 0.046, α = 0.05). Because residues Gly^P710^/Gln^P710^/Arg^P710^, Glu^P729^ and Lys^P930^ are mainly appeared with the phenotype of SC-EV71 strains, here we considered these residues as virulent determinants.

When the virulent determinants were used as the criteria to identify SC-EV71 strains, 15 from 25 SC-EV71 strains were correctly identified, the sensitivity was 60%. The SC-EV71 strains isolated from Malaysia, Japan, Singapore and USA have the highest sensitivity (100.0%), while the SC-EV71 strains from Taiwan, Australia and South Korea have a lower sensitivity (0∼33.3%) (Table 3). When these virulent determinants were used as the criteria for the identification of MC-EV71 strains, 24 from 31 MC-EV71 strains were correctly identified, the specificity was 77.4%. The MC-EV71 strains isolated from China, Norway, Australia and South Korea have the highest specificity (92.3%∼100.0%). The MC-EV71 strains from Taiwan, Malaysia and Singapore have a lower specificity (50%∼75.0%)(Table 3).

**Table 3:** Sensitivity and specificity for the identification of SC-EV71 and MC-EV71 strains according to P710/P729/P930 in polyprotein and P272/P488/P700 in 5′-NTR.

country	SC-EV71 strains			MC-EV71 strains		
	number of strains	sensitivity of 3 positive position in polyprotein	sensitivity of 3 positive position in 5′-NTR	number of strains	specificity of 3 positive position in polyprotein	specificity of 3 positive position in 5′-NTR
Malaysia	6	100.0%	100.0%	7	57.1%	28.6%
Japan	1	100.0%	100.0%	0	-	-
Taiwan	5	20.0%	60.0%	4	75.0%	0.0%
Singapore	1	100.0%	100.0%	4	50.0%	0.0%
South Korea	1	0.0%	0.0%	1	100.0%	100.0%
Australia	3	33.3%	100.0%	1	100.0%	100.0%
Norway	0	-	-	1	100.0%	100.0%
USA	2	100.0%	100.0%	0	-	-
China	6	50.0%	16.7%	13	92.3%	100.0%
total	25	60.0%	68.0%	31	77.4%	58.1%

### Analysis of the nucleotide sequence of 5′-NTR and 3′-NTR

To analyze the variation of 5′-NTR and 3′-NTR, the complete genomic sequences of 56 EV71 strains were aligned. Among about 740 nucleotide positions of 5′-NTR, residues in 202 positions vary obviously.

According to the results of SPSS analysis, four bases on three positions (G^P272^, U^P488^ and A^P700^/U^P700^) were more likely appeared in SC-EV71 strains than in MC-EV71 strains and were identified to be correlated with virulent phenotype. When the four amino acids were considered as virulent determinants to identify virulent phenotype of the 56 EV71 strains, 17 from 25 SC-EV71 strains were correctly identified; the sensitivity is 68.0%. In Table 3, the SC-EV71 strains isolated from Malaysia, Japan, Singapore, Australia and USA have the highest sensitivity(100.0%), but the SC-EV71 strains from China and South Korea have the lowest sensitivity (0∼16.7%). Taiwan has the middle sensitivity of 60%. This result is basically consistent with result based on polyprotein. In MC-EV71 strains, 18 from 31 non-virulent strains were correctly identified; the specificity is 58.1%. The MC-EV71 strains from China, Norway, Australia and South Korea have the highest specificity (100.0%). The MC-EV71 strains from Singapore, Malaysia and Taiwan have the lowest specificity (0.0%∼28.6%).

Through fragment base content analysis, twenty fragments (F1∼F20) were obtained. There are 10 nucleotide sites in each fragment except fragment F20 (12 nucleotide sites). The constituent ratios of the four bases (A, U, G and C) between SC-EV71 and MC-EV71 strains showed significant difference in F19. The difference of constituent ratios of base A (24.4% vs 19.1%) and C (35.2% vs 29.1%) between SC-EV71 and MC-EV71 strains in F19 are the highest in all fragments, and the difference of the constitution ratios of base G (18.0% vs 23.3%) and U (22.4% vs 28.5%) between SC-EV71 and MC-EV71 strains in this fragment are the lowest. F19 covers the region from 696 to 714 bp in BJ08-Z004-3, and the A^P700^/U^P700^ mentioned above also located in this region.

Analysis of the 83 nucleotides in 3′ -NTR showed no virulence associated nucleotides.

### Comparison of the virulent determinants of polyprotein and 5′-NTR in identification of virulent strains

In 25 SC-EV71 strains, total 15 strains were recognized by either Gly^P710^/Gln^P710^/Arg^P710^, or Glu^P729^, or Lys^P930^ in polyprotein. Six of the 15 strains were recognized by the virulent determinants at all of the three positions; four strains were recognized by both Glu^P729^ and Lys^P930^, four strains by Gly^P710^/Gln^P710^/Arg^P710^, and one strain by only Lys^P930^.

When identified by virulent determinants in 5′-NTR, 17 SC-EV71 strains were recognized by either G^P272^, or U^P488^, or A^P700^/U^P700^. Eleven of them were determined by the virulent determinants at all the three positions, six of them were determined only by U^P488^.

Although the sensitivity for recognizing SC-EV71 strains by virulent determinants in 5′-NTR is higher than that of polyprotein, the specificity for identifying MC-EV71 strains by 5′-NTR is lower than polyprotein (Table 3).

In 25 SC-EV71 strains, total 19 strains were recognized by either the virulent determinants of polyprotein or those of 5′-NTR. 13 strains were recognized by both of them. Among the 6 other recognized strains, 4 strains isolated from Tainan in Taiwan (1998)_and Australia (1999) were only recognized by those of 5′-NTR, 2 strains isolated from Shenzen in China (2008) were only recognized by those of polyprotein.

### 3D structure of VP1 and protease 2A

For picornaviruses, such as poliovirus and human rhinovirus, the surfaces of these viruses have a prominent star-shaped plateau (mesa), which is surrounded by a deep depression (‘canyon’). Beneath the canyon floor is the hydrophobic pocket. VP1 is the main part to constitute canyon and hydrophobic pocket. Since P710 and P729 located in VP1, we predicted the 3D structure of VP1 to see where the two sites are and what influences the amino acid variations may make on the two sites.

**Figure 2:**
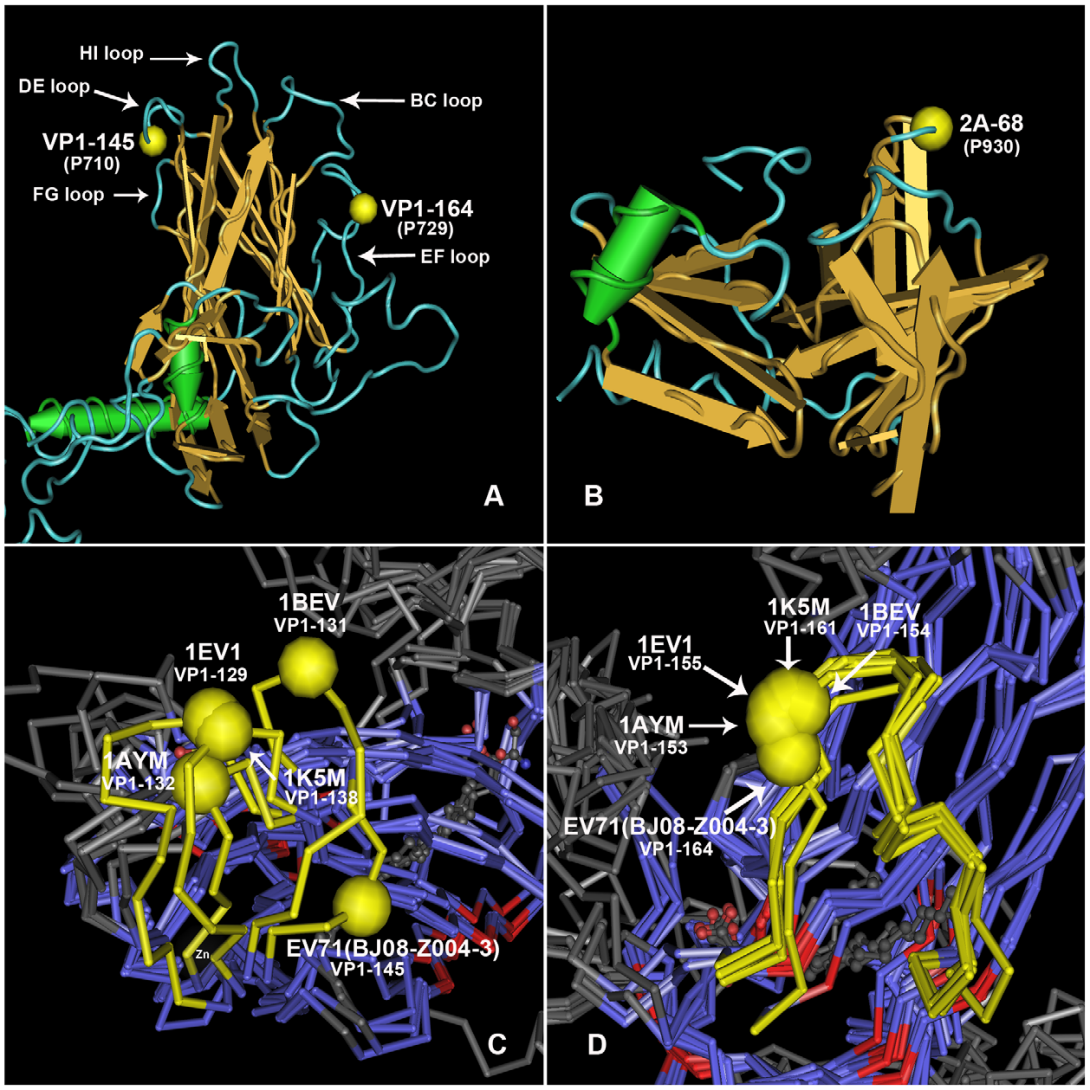
The predicted three dimensional structures of VP1 protein and protease 2A of EV71. A: predicted three dimensional structure of VP1 protein. The left yellow ball represents the 145^th^ amino acid residue (P710) at VP1, and the right one represents the 164^th^ residue (P729) at VP1. B: predicted three dimensional structure of Protease 2A protein. The yellow ball represents the 68^th^ residue (P930) at 2A protein. C: the comparison of DE loop between predicted VP1 structure of EV71 (BJ08-Z004-3) and the real structure of 4 other picornaviruses. The yellow tubes represent the DE loop, and the yellow balls represent the corresponding residue on each strain to the residue on VP1-145 of EV71. D: the comparison of EF loop between predicted VP1 structure of EV71 and the real structure of 4 other picornaviruses. The yellow tubes represent the EF loop, and the yellow balls represent the corresponding residue on each strain to the residue on VP1-164 of EV71.

From the predicted 3D structure of EV71 VP1 protein (Figure 2A), we can see that P710 (VP1-145) and P729 (VP1-164) were located in the DE loop and EF loop respectively. Both DE loop and EF loop located at the surface of canyon and not at the deep hydrophobic pocket. We further blasted the structure of VP1 with other picornaviruses by VAST, including Echovirus I (PDB ID: 1EV1), Bovine enterovirus (PDB ID: 1BEV), human rhinovirus 14 (PDB ID: 1K5M) and human rhinovirus 16 (PDB ID: 1AYM), the dimensional configurations of DE loop, just like BC loop, is in diversiform (Figure 2C). Contrary to the high dimensional structural variations of DE loop, the variations of EF loop are low and the dimensional structure is relatively stable in these picornaviruses (Figure 2D).

Protease 2A is one of the picornaviral proteinases responsible for cleavage of the host cell protein eIF4G, polyprotein and maybe dystrophin, a key protein component of heart muscle that is frequently mutated in inherited forms of dilated cardiomyopathy. P930 located in Protease 2A. From the predicted protein structure of Protease 2A (Figure 2B), we can see that P930 located on the 68^th^ of Protease 2A, which is on the surface of the protease.

### Comparison of the predicted secondary structures of consensus 5′-NTR sequence between SC-EV71 and MC-EV71 strains

The predicted secondary structures of the consensus 5′-NTR nucleotide sequences of 31 MC-EV71 and 25 SC-EV71 strains were constructed by RNAStructure program and RNAalifold server respectively. Here we mainly present the results of RNAStructure and the results were then verified by RNAalifold server. From the structure we found that the energy of SC-EV71 stains (−265.6kcal/mol) is lower than MC-EV71 strains (−257.6 kcal/mol). To find the factors for this difference, a further comparison on the consensus nucleotide sequence was performed. There are 12 positions with obvious nucleotide substitutions. One position (P63) is in cloverleaf, three (P100, P103 and P106) in domain I, four (P278, P282, P302 and P408) in domain II, two (P490 and P504) in domain III, and two (P713 and P722) in domain V. The position we mentioned in the bracket refers to the consensus sequence of SC-EV71 strains.

**Figure 3:**
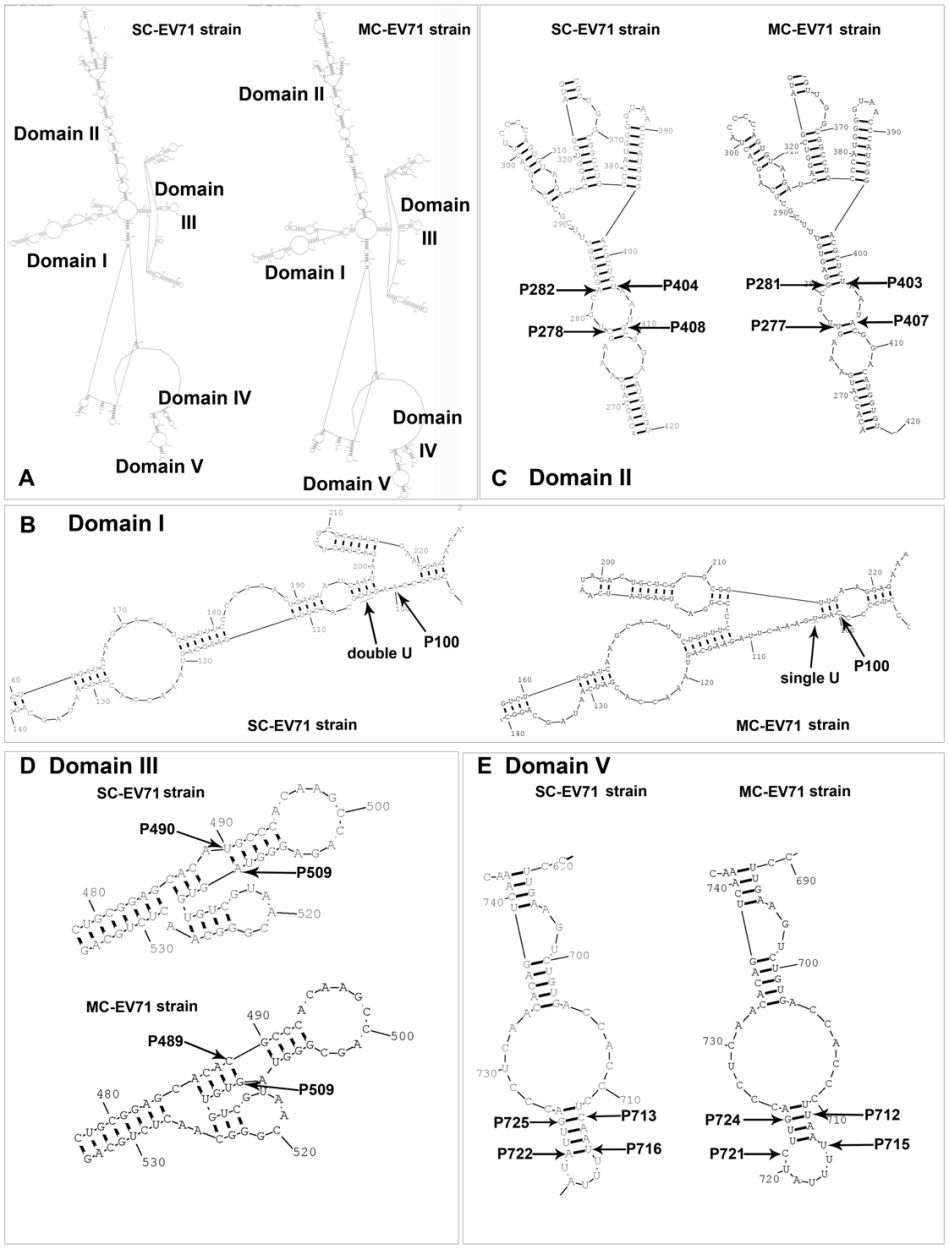
The comparison of 5′NTR secondary structure predicted by RNAStructure between the SC-EV71 and MC-EV71 strain. A: the whole structural comparison. B: the comparison of specific part of Domain I. C: the comparison of specific part of Domain II. D: the comparison of specific part of Domain III. E: the comparison of specific part of Domain V.

At position P100 in the consensus sequence of SC-EV71 strains, base C in MC-EV71 strains was substituted by A and an additional U was inserted into the position P102 and P104, which resulted in the reconstruction of domain I and one more base pair was formed (Figure 3B).

In domain II base U^P277^ in the consensus sequence of MC-EV71 strains was substituted by base C^P278^ in SC-EV71 strains, and base A^P407^ in MC-EV71 strains was substituted by base G^P408^ in SC-EV71 strains simultaneously. The more stable base pair (C^P278^–G^P408^) was formed in SC-EV71 strains instead of base pair (U^P277^–A^P407^) in MC-EV71 stains. Furthermore, base G^P281^ in MC-EV71 strains was substituted by base A^P282^ in SC-EV71 strains, then a higher stable base pair A^P282^–U^P404^ but not the base pair G^P281^·U^P403^ was formed (Figure 3C). So the domain II in 5′-NTR of SC-EV71 strains is more stable than that of MC-EV71 strains.

In domain III, a base pair C^P489^–G^P509^ in MC-EV71 strains was substituted by U^P490^–A^P509^ in SC–EV71 strains. Although the energy of the secondary structure becomes higher after GC base pair was substituted by AU, the sub stem-loop on the main stem of domain III was reconstructed and one more base pair was formed in SC-EV71 strains (Figure 3D).

In domain V, a more stable base pair C^P713^–G^P725^ instead of the U^P712^·G^P724^ in MC–EV71 strains was formed in SC-EV71 strains, and an additional base pair A^P722^–U^P716^ was formed in SC-EV71 strains (Figure 3E).

The results predicted by RNAStructure were verified by RNAalifold Server. We found results of Domain ? are consistent by the two programs.

## Discussion

The infection of EV71 can be asymptomatic or can cause mild clinical symptoms, such as diarrhea, rashes, herpangina, vesicular lesions on the hands, feet, and oral mucosa and so on. But some EV71 infection can also cause severe clinical symptoms, such as acute flaccid paralysis mimicking paralytic poliomyelitis, bulbar and brainstem encephalitis, the Guillain–Barré syndrome [18], [19] and rapidly fatal pulmonary edema and hemorrhage [20]. To look for virulent determinants at the molecular level, besides the five strains isolated in our laboratory; another 51 strains with different virulence and clear backgrounds from the Genebank database were included in our study.

We mapped Gly^P710^/Gln^P710^/Arg^P710^, Glu^P729^ on the capsid protein VP1, Lys^P930^ on protease 2A and G^P272^, U^P488^ and A^P700^/U^P700^ on the 5′-NTR, are associated with virulent phenotype. Using these virulent determinants as criteria to identify SC-EV71 strains, most of the SC-EV71 strains (19/25) can be identified. The sensitivity is high. However, disparity of sensitivity was found when EV71 strains from different countries were analyzed (Table 3). We analyzed the unrecognized SC-EV71 strains and found the one strain in South Korea was classified as C3 subgenotype and the other unrecognized SC-EV71 strains were classified as C4 subgenotype. By far, C4 subgenotype EV71 viruses were only detected in the mainland of China and Taiwan. So it seems that C4 SC-EV71 strains can't be well recognized by the virulent determinants we found, which work well in other subgenotypes.

Resolution of the structures of some picornaviruses, such as poliovirus and human rhinovirus, revealed that the surfaces of these viruses have a corrugated topography; there is a prominent star-shaped plateau (mesa) at the 5-fold axis of symmetry, surrounded by a deep depression (‘canyon’). The canyon is proved to be a receptor-binding site. Beneath the canyon floor is the hydrophobic pocket, which is the binding site for lipids or anti-picornavirus drugs. VP1 is the main part to constitute canyon and hydrophobic pocket. Variations of amino acids on VP1 especially on the inter-surface of canyon can likely influent the binding of virus to the receptor, and thus influent virus pathogenicity. P710 and P729 located in DE loop and EF loop of VP1, which are located at the surface of canyon. The high diversification of DE loop supports the assumption that it may participate in the interaction with receptor or neutralizing antibody. In our analysis, on DE loop the amino acid substitution is acidic amino acid Glu in MC-EV71 strains replaced by acyclic amino acid Gly, amidic amino acid Gln or alkaline amino acid Arg in P710 in SC-EV71 strains. These substitutions certainly will change the biochemical characteristics of DE loop and thus influence the virus binding to antibody or receptor, thereby affecting the virulence of the virus. Consistent with our results, some studies proved that the amino acid substitution of the Gly to Glu at position P710 of VP1 could increase EV71 virulence in mouse [21], [22]. Our results indicates the amino acid substitution at position P710 from Glu to Gly/Gln/Arg will enhance the virulence in human, so the mice model which extensively adopted to prove the virulence maybe not the suitable animal model for this investigation. We also found besides Glycine, Glu or Arg substitution at position P710 is possible to enhance EV71 virulence in humankind.

Amino acid substitution on EF loop in P729 is acidic amino acid Asp replaced by another acidic amino acid Glu. Since this substitution did not change the polarity of the amino acids, we speculate substitution of P729 (VP1-164) may not result in the virus structure variation and can only influence the binding effect to antibody or receptor slightly.

Protease 2A is responsible for cleavage of the host cell protein eIF4G, polyprotein and maybe dystrophin, a key protein component of heart muscle that is frequently mutated in inherited forms of dilated cardiomyopathy. Protease 2A also leads to induction of apoptosis in poliovirus infectious cells. In SC-EV71 strains on P930, sulfur-containing amino acid (Met) or basic amino acid (Arg) were replaced by basic amino acid (Lys), this replacement may influence the activity of protease.

In previous study, mutations in the internal ribosomal entry site (IRES) within the 5′-NTR, especially of poliovirus, have been shown to be correlated with neurovirulence [23]. In the linear nucleotide sequence of 5′-NTR of EV71, four bases on three positions (G^P272^, U^P488^ and A^P700^/U^P700^) were found to be correlated with virulent phenotype. G^P272^ and U^P488^ just located in the IRES region. The predicted secondary structure further proved that the mutation of base C at position 488 in strain BJ08-Z004-3 (position 491 in BrCr) to base U can cause the reconstruction of Domain III. In SC-EV71 strains, a sub-stem-loop flanking the main stem of Domain III may be constructed with one more base pair (U-A) to form a longer stem (Figure 3D).

At the present stage, there are some differences between RNA structures predicted by predicted software and the actual structure. Moreover the RNA sequences are different among each others, predicted secondary structures based on consensus sequence can reflect the structure characters of a class of strains, however the precondition is that nucleotides in this region is relatively conservative. In this condition consensus sequence can actually reflect the characters of most of the strains. However for the highly variable 5′-NTR, especially Domain V, this approach may lead to large errors. To reduce these errors, we also used fragment base content analysis for 5′-NTR as complementary to the two methods. We found that in the fragment F19 (at 696 −714 bp in 5′-NTR), there is significant difference in the nucleotide constitution ratios between SC-EV71 and MC-EV71 strains. The constitution ratio of A/C is higher and G/U is lower in SC-EV71 strains than MC-EV71 strains. The A^P700^/U^P700^ which we found may be associated with virulent phenotype also located in this region.

In conclusion, we analyzed the genomes of 56 EV71 strains and found Gly^P710^/Gln^P710^/Arg^P710^, Glu^P729^ in VP1, Lys^P930^ in protease 2A and G^P272^, U^P488^ and A^P700^/U^P700^ in 5′-NTR are correlated with the virulence of EV71. It is the first time to analyze the genome at such a comprehensive level. The amino acid or nucleotide variations at the positions we found may influence the combination ability of virus to the host, virus RNA translation efficiency and the enzymatic activity of protease.

## Materials and Methods

### Ethics statement

This study has obtained ethics approval from the ethics committee at Beijing center for disease control and prevention. We obtained written informed consent from the parents of all the children involved in our study.

### Viruses and Cells

Five EV71 strains were isolated and sequenced in our laboratory (Table S1). Three of these strains BJ08/Z011/4, BJ08/Z025/5 and BJ08/Z004/3 were isolated from skin vesicle fluids collected from mild HFMD patients (with blisters only), one strain BJ08/Z020/1 was from skin vesicle fluid collected from severe HFMD patients (with CNS symptoms), and one strain BJ06-SJS06 from stool sample collected from healthy child. Additional 51 EV71 strains (Table S1) with the whole genome sequence and known backgrounds, especially the clinical features, were downloaded from Genbank database and enrolled in this study.

Those EV71 strains isolated from patients with CNS associated symptoms, such as encephalitis, neurovirulent, pulmonary oedema, (aseptic) meningitis, acute cardiogenic shock, poliomyelitis-like paralysis, or patients with severe HFMD, death, are all considered as EV71 strains from severe cases (SC-EV71); those strains from mild HFMD, HFMD, healthy infants are considered as EV71 strains from mild cases (MC-EV71).

Human rhabdomyosarcoma (RD) cells provided by National Polio Laboratory of Chinese center for disease control and prevention were used for EV71 isolation. RD cells were maintained in MEM (GIBCO) supplemented with 10% FBS (GIBCO) and 100 µg/ml streptomycin sulfate (GIBCO).

### Virus isolation and RNA extraction

Stool samples were fully vibrated into fecal suspensions with PBS containing 10% chloroform, and then centrifuged at 4000 rpm/min for 20 minutes. Supernatants were aspired and added to monolayer of RD cells. Throat swabs were directly added to RD cell monolayers. Negative cell controls were set simultaneously. When characteristic enterovirus cytopathic effects (CPE) appeared, the cultures were stored at −20°C for a second passage. The second passage material was pooled for real-time RT-PCR. If no CPE appeared after 7 days, a blind passage was performed for a further 7 days. If still no CPE, the sample was considered negative.

Virus RNA was extracted from 140 µl virus fluids with the QIAamp Viral RNA Kit (Qiagen). RNA was eluted with 60 µl AVE.

### RT-PCR amplification and sequencing

Primers used for amplification of the complete genome sequences are synthesized by Sangon Biotech (Shanghai) Co. Ltd. and listed in Table S2. All RT-PCRs were carried out with OneStep RT-PCR Kit (Qiagen), and 3 µl of each primer (10 µM) in a total volume of 50 µl. After reverse transcription consisting of 50°C for 30 min, then the initial PCR activation step at 95°C for 15 min, there were 35 cycles of amplification consisting of 94°C for 30 s, followed by annealing at 52°C for 30 s and extension of 72°C for 120 s, with a final extension of 72°C for 10 min. Correctly sized PCR products were directly sequenced bi-directionally with BigDye 3.1 chemistry on an ABI 3100 sequencer. The sequencing reactions were performed with the same amplification primers as used in the RT-PCR.

The sequences of 5′-NTR and 3′-NTR in 4 EV71 strain (FJ606447.1, FJ606448.1, FJ606449.1, and FJ606450.1) were sequenced by Beijing Genomics Institute using 5′-full race kit and 3′-full race core set ver. 2.0 (TakaRa).

### Sequence Assembly and Nucleotide sequence accession numbers

Sequence data for each isolate was formatted and compiled into contiguous segments by Editseq and Seqman program of DNASTAR Software (version 5.0).

The nucleotide sequences of the five EV71 strains were submitted to the Genbank database under accession numbers HQ129932.1, FJ606447.1, FJ606448.1, FJ606449.1 and FJ606450.1 for complete genome sequences.

### Phylogenetic analysis

The VP1 protein coding regions and complete polyprotein coding regions were compared to each other with Bioedit software (version 7.0.9.0) program. Phylogenetic trees were constructed by neighbor-joining method with MEGA software (version 4.0). Evolutionary distances were estimated by Maximum Composite Likelihood (MCL) method. The reliability of the neighbor-joining tree was estimated by bootstrap analysis with 1000 pseudo replicate data sets, and the bootstrap values of over 80% supporting each cluster are shown at the nodes. The reference Coxsackie A16 G-10 Strain (CA16-G-10) was included in the analysis as an out group.

### Analysis of deduced amino acids in polyprotein and nucleotides in 5′-NTR and 3′-NTR

The genomes of 25 SC-EV71 and 31 MC-EV71 strains were aligned using ClustalW multiple alignment tools (Bioedit program version 7.0.5.1), then deduced amino acid sequences of polyprotein, nucleotides in 5′-NTR and 3′-NTR in all of the strains were aligned. The constituent ratio of each deduced amino acid in polyprotein or nucleotides in 5′-NTR and 3′-NTR at each variable position between SC-EV71 and MC-EV71 strains was compared with Chi-square and Fisher's exact test by SPSS (version 13.0). Level of significance (α) was set at 0.05.

### Fragment Base Content Analysis

As the variation of nucleotides in 5′-NTR was larger than that in polyprotein, a new method was carried out in the process of analysis. Nucleotides on the position where nucleotides are different in more than two strains were selected from each strain and connected to form new nucleotide sequences. Then the new sequences were segmented into short fragments (10 positions per fragment). The constituent ratios of base A/C/G/U in each fragment were calculated and compared between SC-EV71 and MC-EV71 strains.

### Prediction of 3-Dimensional construction of virulent associated proteins and second structure of 5′-NTR

The three-dimensional structures of SC-EV71 correlated proteins were predicted by robetta server (http://robetta.bakerlab.org/submit.jsp). The predicted models were viewed in Cn3D (version 4.1) (http://www.ncbi.nlm.nih.gov/Structure/CN3D/cn3d.shtml) and compared with the structure of other closely related enteroviruses (http://www.ncbi.nlm.nih.gov/Structure/VAST/vastsearch.html).

The consensus sequence of SC-EV71 strains and MC-EV71 strains were performed by RNAalifold server in Vienna RNA webservers (http://rna.tbi.univie.ac.at/) [24], [25], [26]. The predicted secondary structures of 5′-NTR were constructed by RNAalifold server and RNASructure (version 5.2) (http://rna.urmc.rochester.edu/RNAstructure.html).

## Supporting Information

Table S1 Backgrounds of the 56 EV71 strains enrolled in this study. * : inferred from the nomenclature of EV71 virus isolated from Malaysia.
(DOC)

Table S2 Oligonucleotide primers used for genome sequencing of the five EV71 strains isolated in our lab.
(DOC)
