# Supplementary material for: Molecular Analysis of Virulent Determinants of Enterovirus 71
Source: PLoS One. 2011 Oct 19;6(10):e26237. doi: 10.1371/journal.pone.0026237 (PMC3198388; doi:10.1371/journal.pone.0026237)
Supplement: Table S1 — Backgrounds of the 56 EV71 strains enrolled in this study. * : inferred from the nomenclature of EV71 virus isolated from Malaysia. (DOC) [file pone.0026237.s001.doc]

| GenBank accession no. | Isolate | Clinical features | Isolated Year | Origin | Reference |
| --- | --- | --- | --- | --- | --- |
| FJ606449.1 | BJ08/Z020/1 | Severe HFMD | 2008 | China | No |
| FJ606448.1 | BJ08/Z011/4 | HFMD | 2008 | China | No |
| FJ606450.1 | BJ08/Z025/5 | HFMD | 2008 | China | No |
| FJ606447.1 | BJ08/Z004/3 | HFMD | 2008 | China | No |
| HQ129932.1 | BJ06-SJS06 | Healthy child | 2008 | China | No |
| ETU22521.1 | BrCr | encephalitis | 1970 | USA | [1,2] |
| ETU22522.1 | MS/7423/87 | neurovirulent | 1987 | USA | [2] |
| AF136379.1 | NCKU9822 | neurovirulence Death | 1998 | Taiwan | [3] |
| AB469182.1 | SKEV006/Malaysia/97 | fatal encephalitis | 1997 | Malaysia | [4] |
| AF316321.2 | 5865/sin/000009 | encephalitis,interstitial pneumonitis and pulmonary oedema | 2000 | Singapore | [5] |
| AF304458.1 | Tainan/4643/98 | aseptic meningitis | 1998 | Taiwan | [6] |
| DQ341367.1 | MY821/3/SAR/97 | meningitis | 1997 | Malaysia | [7] |
| DQ341368.1 | MY104/9/SAR/97 | acute cardiogenic shock | 1997 | Malaysia | [7] |
| DQ381846.1 | 6F/AUS/6/99 | brainstem encephalitis/myelitis | 1999 | Australia | [7] |
| DQ341357.1 | 7F/AUS/6/99 | meningitis | 1999 | Australia | [7] |
| EU364841.1 | 26M/AUS/4/99 | neurovirulent | 1999 | Australia | [8] |
| DQ341366.1 | SB2864/SAR/00 | poliomyelitis-like paralysis | 2000 | Malaysia | [7] |
| AF119795.2 | TW/2272/98 | Died of pulmonary hemorrhage and shock | 1998 | Taiwan | [9] |
| AB482183.1 | Nagoya | HFMD with aseptic meningitis | 1973 | Japan | [10] |
| DQ133458.1 | 984 | pediatric patient with encephalitis | 2004 | Taiwan | Genbank |
| DQ133459.1 | 1235 | pediatric patient with encephalitis | 2004 | Taiwan | Genbank |
| FJ607338.1 | 605/SHENZHEN/08/China/HFMD Severe/2008 | HFMD Severe | 2008 | China | Genbank |
| FJ607337.1 | 121/SHENZHEN/08/China/HFMD Fatal/2008 | HFMD Fatal | 2008 | China | Genbank |
| AF352027.1 | 5666/sin/002209 | mild HFMD | 2000 | Singapore | [5] |
| AF119796.3 | TW/2086/98 | mild HFMD | 1998 | Taiwan | [9] |
| FJ439769.1 | Fuyang/0805 | HFMD | 2008 | China | [11] |
| DQ341354.1 | 3799/SIN/98 | HFMD | 1998 | Singapore | [7] |
| AF304457.1 | Tainan/5746/98 | HFMD | 1998 | Taiwan | [12] |
| AF304459.1 | Tainan/6092/98 | HFMD | 1998 | Taiwan | [12] |
| DQ341358.1 | S40221/SAR/00 | HFMD | 2000 | Malaysia | [7] |
| DQ341359.1 | S10862/SAR/98 | HFMD | 1998 | Malaysia | [7] |
| DQ341361.1 | 1M/AUS/12/00 | HFMD | 2000 | Australia | [7] |
| AF176044.1 | 1245a/98/tw | HFMD | 1998 | Taiwan | Genbank |
| AF302996.1 | SHZH98 | HFMD | 1998 | China | [13] |
| FJ607335.1 | 4/SHENZHEN/08/China/HFMD/2008 | HFMD | 2008 | China | Genbank |
| FJ607334.1 | 1/SHENZHEN/08/China/HFMD/2008 | HFMD | 2008 | China | Genbank |
| FJ607336.1 | 28/SHENZHEN/08/China/HFMD/2008 | HFMD | 2008 | China | Genbank |
| DQ341364.1 | 5511/SIN/00 | HFMD | 2000 | Australia | [7] |
| GQ994988.1 | Anhui1/09/China | HFMD with symptoms of CNS involvement | 2009 | China | [14] |
| GQ994989.1 | Chongqing1/09/China | mild HFMD | 2009 | China | [14] |
| GQ994990.1 | Chongqing2/09/China | mild HFMD | 2009 | China | [14] |
| GQ994991.1 | Chongqing3/09/China | mild HFMD | 2009 | China | [14] |
| GQ994992.1 | Henan2/09/China | HFMD with symptoms of CNS involvement | 2009 | China | [14] |
| GU196833.1 | Henan1/09/China | HFMD with symptoms of CNS involvement | 2009 | China | [14] |
| AJ586873.1 | 9/97/SHA89 | HFMD Fatal | 1997 | Malaysia | [15] |
| DQ341355.1 | 06/KOR/00 | aseptic meningitis | 2000 | South Korea | [13] |
| DQ341356.1 | 03/KOR/00 | HFMD | 2000 | South Korea | [13] |
| DQ452074.1 | 804/NO/03 | healthy infants | 2003 | Norway | [16] |
| FJ828519.1 | BJ08 | HFMD | 2008 | China | [17] |
| FJ461781.1 | NUH0083/SIN/08 | HFMD | 2008 | Singapore | Genbank |
| DQ341363.1 | S19841/SAR/03 | HFMD | 2003^*^ | Malaysia | Genbank |
| AM396584.1 | ENT/PM/SHA52 | HFMD | 1997 | Malaysia | [15] |
| AM396585.1 | ENT/PM/SHA71 | HFMD | 1997 | Malaysia | [15] |
| AM396586.1 | SAR/SHA66 | HFMD | 1997 | Malaysia | [15] |
| AM396588.1 | SAR/SHA63 | HFMD | 1997 | Malaysia | [15] |
| AM396587.1 | UH1/PM/1997 | fatal | 1997 | Malaysia | [15] |

^*^ : inferred from the name of other virus isolated from Malaysia

1. Brown BA, Oberste MS, Alexander JP, Jr., Kennett ML, Pallansch MA (1999) Molecular epidemiology and evolution of enterovirus 71 strains isolated from 1970 to 1998. J Virol 73: 9969-9975.

2. Brown BA, Pallansch MA (1995) Complete nucleotide sequence of enterovirus 71 is distinct from poliovirus. Virus Res 39: 195-205.

3. Yan JJ, Wang JR, Liu CC, Yang HB, Su IJ (2000) An outbreak of enterovirus 71 infection in Taiwan 1998: a comprehensive pathological, virological, and molecular study on a case of fulminant encephalitis. J Clin Virol 17: 13-22.

4. Yamayoshi S, Yamashita Y, Li J, Hanagata N, Minowa T, et al. (2009) Scavenger receptor B2 is a cellular receptor for enterovirus 71. Nat Med 15: 798-801.

5. Singh S, Poh CL, Chow VT (2002) Complete sequence analyses of enterovirus 71 strains from fatal and non-fatal cases of the hand, foot and mouth disease outbreak in Singapore (2000). Microbiol Immunol 46: 801-808.

6. Wang JR, Tuan YC, Tsai HP, Yan JJ, Liu CC, et al. (2002) Change of major genotype of enterovirus 71 in outbreaks of hand-foot-and-mouth disease in Taiwan between 1998 and 2000. J Clin Microbiol 40: 10-15.

7. McMinn P, Lindsay K, Perera D, Chan HM, Chan KP, et al. (2001) Phylogenetic analysis of enterovirus 71 strains isolated during linked epidemics in Malaysia, Singapore, and Western Australia. J Virol 75: 7732-7738.

8. McMinn P, Stratov I, Nagarajan L, Davis S (2001) Neurological manifestations of enterovirus 71 infection in children during an outbreak of hand, foot, and mouth disease in Western Australia. Clin Infect Dis 32: 236-242.

9. Shih SR, Ho MS, Lin KH, Wu SL, Chen YT, et al. (2000) Genetic analysis of enterovirus 71 isolated from fatal and non-fatal cases of hand, foot and mouth disease during an epidemic in Taiwan, 1998. Virus Res 68: 127-136.

10. Shimizu H, Utama A, Yoshii K, Yoshida H, Yoneyama T, et al. (1999) Enterovirus 71 from fatal and nonfatal cases of hand, foot and mouth disease epidemics in Malaysia, Japan and Taiwan in 1997-1998. Jpn J Infect Dis 52: 12-15.

11. Wu Z, Yang F, Zhao R, Zhao L, Guo D, et al. (2009) Identification of small interfering RNAs which inhibit the replication of several Enterovirus 71 strains in China. J Virol Methods 159: 233-238.

12. Yan JJ, Su IJ, Chen PF, Liu CC, Yu CK, et al. (2001) Complete genome analysis of enterovirus 71 isolated from an outbreak in Taiwan and rapid identification of enterovirus 71 and coxsackievirus A16 by RT-PCR. J Med Virol 65: 331-339.

13. Cardosa MJ, Perera D, Brown BA, Cheon D, Chan HM, et al. (2003) Molecular epidemiology of human enterovirus 71 strains and recent outbreaks in the Asia-Pacific region: comparative analysis of the VP1 and VP4 genes. Emerg Infect Dis 9: 461-468.

14. Chang GH, Lin L, Luo YJ, Cai LJ, Wu XY, et al. Sequence analysis of six enterovirus 71 strains with different virulences in humans. Virus Res 151: 66-73.

15. Bible JM, Iturriza-Gomara M, Megson B, Brown D, Pantelidis P, et al. (2008) Molecular epidemiology of human enterovirus 71 in the United Kingdom from 1998 to 2006. J Clin Microbiol 46: 3192-3200.

16. Witso E, Palacios G, Ronningen KS, Cinek O, Janowitz D, et al. (2007) Asymptomatic circulation of HEV71 in Norway. Virus Res 123: 19-29.

17. Yao X, Mao QY, Huang WJ, He P, Zhou C, et al. (2009) [Genetic characterization of enterovirus 71 complete genome isolated in Beijing, 2008]. Zhonghua Liu Xing Bing Xue Za Zhi 30: 729-732.
