# Supplementary material for: Molecular Analysis of Virulent Determinants of Enterovirus 71
Source: PLoS One. 2011 Oct 19;6(10):e26237. doi: 10.1371/journal.pone.0026237 (PMC3198388; doi:10.1371/journal.pone.0026237)
Supplement: Table S2 — Oligonucleotide primers used for genome sequencing of the five EV71 strains isolated in our lab. (DOC) [file pone.0026237.s002.doc]

| Segment | Primer | Position | sequence |
| --- | --- | --- | --- |
| A | A-u | 1-20 | TTAAAACAGCCTGTGGGTTG |
|  | A-d | 1503-1481 | AAATTAATCCACTGGTGTGGGCA |
| B | B-u | 1175-1197 | AAGGGATGGTACTGGAAGTTCCC |
|  | B-d | 2529-2551 | TCGATGACTGCTCACCTGTGTGT |
| C | C-u | 2357-2379 | GCACTAGCGGCAGCCCAAAAGAA |
|  | C-d | 3791-3816 | AAAGCATCTCCGAGACCCTTGATGTA |
| D | D-u | 3635-3657 | GGTCACTCAGAACCTGGTGATTG |
|  | D-d | 5110-5136 | AGGAGATCGCTAATAGCGTCTGGGGCT |
| E | E-u | 4910-4935 | AAACGTTGCAGCCCATTAGTGTGTGG |
|  | E-d | 6395-6420 | AGCTCGTCCTTGACATAAGTGGAGTA |
| F | F-u | 6218-6243 | ATGAGCATGGAGGAGGCCTGCTACGG |
|  | F-d | 7382-7405 | GCTATTCTGGTTATAACAAATTTA |
